# Analysis of the impact of clinical practices on salivary biomarkers of inflammation and stress in oral surgery postgraduate students: a pilot study

**DOI:** 10.3389/fmed.2025.1568047

**Published:** 2025-06-13

**Authors:** Laura Barrientos-Moral, María José Gimeno-Longas, Cristina Obispo-Díaz, Andrea Martín-Vacas, Marta Macarena Paz-Cortés, Juan Manuel Aragoneses

**Affiliations:** ^1^Department of Systems Biology, Faculty of Medicine and Health Sciences, Universidad de Alcalá, Alcalá de Henares, Spain; ^2^Experimental and Clinical Dentistry – Research Group, Facultad de Odontología, Universidad Alfonso X El Sabio, Villanueva de la Cañada, Spain; ^3^Department of Cell Biology and Histology, School of Medicine, Universidad Complutense de Madrid, Madrid, Spain; ^4^Clinical Specialties Department, Faculty of Dentistry. Universidad Complutense de Madrid, Madrid, Spain; ^5^Department of Dental Research, Federico Henriquez y Carvajal University, Santo Domingo, Dominican Republic

**Keywords:** dentistry, dental education, oral surgery, inflammation mediators, biomarkers

## Abstract

**Aim:**

To assess through salivary biomarkers if clinical practices generate stress and a systemic inflammatory response in dental surgery post-graduate students.

**Materials and methods:**

A cross-sectional analytical observational study was conducted with students from the Master’s in Clinical Dentistry program. Salivary samples were collected before and after surgical procedures to quantify stress (cortisol) and inflammation biomarkers (IL-6, IL-1β, and CRP). Additionally, students completed the Perceived Stress Scale (PSS) questionnaire prior to the surgical procedure. Descriptive and analytic statistics were conducted with a 95% significance level.

**Results:**

A total analyzed sample included 21 subjects, with a mean age of 25.5 years. The influence of academic year, gender, or smoking status was none found to have a significant impact. The results show a significant decrease in cortisol levels between the pre- and post-measurements (mean difference = −108.2 ± 166.7). However, an increase in IL-6 levels was obtained (*p* < 0.05). High IL-6 levels were associated with elevated CRP levels. An inverse relationship was seen between perceived stress and salivary cortisol concentrations.

**Conclusion:**

The findings show a significant decrease in salivary cortisol (stress) levels and a significant increase in salivary IL-6 levels following a dental implantology procedure in postgraduate oral surgery students.

## 1 Introduction

Stress is defined as a sequence of events that begins with a stimulus (stressor) and culminates in a reaction within the brain, characterized by the perception of stress (1). According to the World Health Organization (WHO), stress is defined as a state of mental tension or worry triggered by challenging situations. It represents a natural response to perceived threats or stimuli, and the way we respond to stress largely determines its impact on our overall well-being (2). When the human body meets the challenge posed by a stressor, a complex response is initiated, involving multiple organs and systems that adapt to address the demand. This process, referred to as the stress response system (SRS), is activated at once following exposure to the stressor through stimulation of the sympathetic nervous system, leading to the secretion of catecholamines by the adrenal medulla. Concurrently, the hypothalamic-pituitary-adrenal (HPA) axis becomes engaged, running at a slower but sustained pace to support the stress response (3). Through a multi-stage cascade, glucocorticoids such as cortisol are released (4). The SRS plays a fundamental role in regulating inflammatory processes, particularly the release of cytokines produced by immune-mediated inflammatory reactions, such as interleukin-1 (IL-1) and interleukin-6 (IL-6) (5, 6), as well as chemokines, adhesion molecules, and acute-phase reactants, which can lead to low-grade inflammation (7–9).

Adrenocortical hormones, or glucocorticoids, have potent anti-inflammatory and immunosuppressive properties. They achieve these effects by inhibiting the production of cytokines and other inflammatory mediators while promoting cytokine resistance. Furthermore, glucocorticoids enhance the acute-phase reaction, a critical part of the body’s immediate response to stress. Cortisol, the principal glucocorticoid, is widely recognized as a key biomarker of stress (10–12). Since cortisol regulates multiple biological systems, dysregulation in its production poses a risk to the individual, as it increases the likelihood of developing stress-related diseases (13). Moreover, it may contribute to the pathophysiology of anxiety and mood disorders (12). As previously noted, cortisol can suppress the production of cytokines, which are molecular signaling glycopeptides that function at remarkably low concentrations to modulate immune responses (14). An increase in pro-inflammatory cytokines and cortisol have been stated in a systematic review with meta-analysis (8), with individual variation according to psychological stressors. Various studies (15–17) have demonstrated this effect evaluating salivary biomarkers. These researches report an inverse relationship between the cortisol response and changes in inflammatory markers in healthy individuals.

A systemic increase in cytokines such as IL-6 and IL-1β in blood serum has been shown to occur when an organism is subjected to stress. Studies evaluating IL-1β levels in blood demonstrate a rapid increase in response to psychosocial stressors, which is associated with inflammatory tissue damage and increased osteoclastic activity (18–22). IL-1β can induce a wide range of behavioural effects in both humans and experimental animals, including anorexia, weight loss, fatigue, somnolence, sleep disturbances, decreased locomotor activity, reduced exploratory behaviour, fear responses, and depression-like syndromes. IL-6, in turn, is involved in multiple physiological functions, particularly those related to immunity. Consequently, elevated IL-6 levels in the bloodstream are associated with various inflammatory-related diseases such as autoimmune disorders (e.g., rheumatoid arthritis, psoriasis), atherosclerosis, cancer, and psychological conditions including depression and anxiety. Individual saliva composition can be influenced by numerous factors, such as age, sex, smoking status, diet, medication, and the pathophysiological condition of the oral cavity (23–25). Moreover, salivary biomarker levels, such as cortisol, in response to an acute stressor can be influenced by interpersonal variables, including each individual’s perception of stress (perceived stress), given that stress is a subjective experience. Additional influencing factors include habituation to the stressor and the functional capacity of the organism’s stress-response system to release substances that aid in coping with the experience. This allows for classification into “responders” and “non-responders” to stress. The cytokines most consistently elevated in saliva in response to acute stress include, are, among others, IL-1β and IL-6 (3). IL-6 is a pro-inflammatory cytokine that stimulates inflammation by activating cytotoxic T cells, producing B cells, complement proteins, and acute-phase proteins. It promotes phagocytosis, increases vascular permeability and cell adhesion, induces the expression of acute-phase proteins, and plays a role in the differentiation of monocytes into macrophages (18, 26). IL-6 is produced by various cell types, including T cells, B cells, and macrophages, among others. It serves as a primary indicator for the release of C-reactive protein (CRP) from the liver as part of the acute-phase response. Although it has both pro- and anti-inflammatory properties, it is typically pro-inflammatory in the context of psychological stress. Also, it has been stated that an increase in IL-6 occurs in saliva after exposing an individual to an acute stressor (8, 16, 18). On the other hand, IL-1β was the first cytokine to be associated with the modulation of neuroendocrine systems in response to stress, particularly the HPA axis. IL-1β may play a significant role in the early phase of the immune response to stress. Studies analyzing IL-1β levels in blood prove a rapid elevation in response to psychosocial stressors (18). IL-1β plays a pivotal role in the inflammatory process, both as a signaling molecule and as an inducer of inflammation. It is strongly associated with inflammatory tissue destruction and is recognized as the agent exhibiting the highest osteoclastic activity among mammalian organisms (19). This cytokine can induce a wide range of behavioral effects in humans and experimental animals such as anorexia, weight loss, lack of energy, drowsiness, sleep disorders, reduced locomotor activity, and depression (20).

The role of glucocorticoids in the suppression of pro-inflammatory cytokine production has been widely proved (16). Under normal physiological conditions, glucocorticoids and cytokines interact closely. For example, cortisol exerts anti-inflammatory effects by inhibiting the transcription of pro-inflammatory cytokines or by activating glucocorticoid receptors, triggering the inhibition of glucocorticoid production by monocytes (11, 27). For this reason, glucocorticoids are often used to suppress acute inflammatory reactions (11).

C-reactive protein is an acute-phase reactant of the innate immune system that is primarily secreted by the liver, but other sources include macrophages, lymphocytes, vascular tissue, or atherosclerotic plaques. The increase in CRP during inflammation results from an increase in CRP-producing cells. It is secreted in response to IL-6 following acute inflammation or injury, promoting the production of pro-inflammatory cytokines. Additionally, it activates the complement cascade (28, 29). Elevated levels of CRP are associated with acute inflammation (29). Salivary and blood CRP levels are particularly similar because, unlike cytokines, CRP reaches the saliva through the bloodstream. This would explain why CRP levels in saliva are more diluted than in blood (9, 18, 30).

Earlier studies have assessed how academic stress can trigger a systemic inflammatory response, such as Lester’s et al study (1), which analyzed changes in salivary cortisol concentrations and their correlation with IL-6 levels in occupational therapy students during the exam period. Similarly, La Fratta et al. (18) analyzed cortisol concentrations and various salivary inflammatory mediators in students over an academic period and how these concentrations changed as the exam period approached. Both authors obtained results showing that academic stress can induce a systemic inflammatory response. However, it has not yet been assessed whether clinical practices in dental surgery postgraduate students, that is, conducting the practical tasks associated with the learning of a profession, can trigger the same response as measured through salivary biomarkers. The aim was to assess through salivary biomarkers if clinical practices generate stress and a systemic inflammatory response in dental surgery post-graduate students.

## 2 Material and methods

An analytical cross-sectional observational study was conducted, following the STROBE guidelines (31). The study was conducted at the Dental Specialties Clinic of Alfonso X El Sabio University (UAX), in Madrid (Spain) between November 2022 and October 2023. Prior to the start of the study, approval was obtained from the Biomedical Research Ethics Committee of the Clínico San Carlos Hospital (code 6.1/22, 22/334-E).

### 2.1 Study sample and sampling procedure

The target population was set up as students of the Master in Oral Surgery, Implants, and Periodontology (OSIP) at UAX (*n* = 24). Inclusion and exclusion criteria were set to refine the study population. The inclusion criteria required participants to hold a degree in Dentistry and be enrolled in the Master in OSIP at UAX, who were about to perform their first placement surgery implant. Exclusion criteria included students who had not signed the informed consent; subjects who refused to participate; students with concomitant condition with inflammatory alterations (endocrine, hormonal, etc.) during the study period; subjects with systemic drugs that affects the inflammatory response or HPA axis (glucocorticoids, steroids, β-blockers, antidepressants, melanin, or any psychoactive drugs) during the study period or in the previous 3 months; students with psychiatric, psychological, neurological disorders; or intraoral inflammatory conditions. Through a non-systematic consecutive cases sampling procedure, we invited all students enrolled in OSIP Master’s program. All subjects invited to take part were provided with informed consent and a patient information sheet. Additionally, their personal data were encoded following the current national and European data protection regulations. After applying the inclusion and exclusion criteria through a health questionnaire, the final sample consisted of 21 students (3 students were excluded), who were provided with the prior instructions for the collection of salivary samples (11, 27, 32–36). Instructions given to participants were:

1. Wash their hands with soap and water prior to salivary collection
2. Refrain from eating or drinking any liquids 60 min before collecting before to avoid contamination from acidic or sugary foods or drinks that could alter the salivary pH
3. Avoid consuming alcohol, tobacco, or systemic medications 12 h prior
4. Avoid rinsing their mouths with water for 10 min
5. Refrain from intense physical exercise or brushing their teeth 45 min prior
6. Not undergoing any dental treatment 24 h prior.

### 2.2 Study procedure

Data from the earlier questionnaire (gender, smoking status, and academic course) were collected as qualitative variables. After reviewing the existing literature on the topic, we designed the study procedure based on studies in which subjects were exposed to psychological/psychosocial stressors. We analyzed how salivary cortisol release is affected both before and after acute stressor exposure (11, 27, 32–36). Furthermore, to analyze how variations in cortisol levels affect the inflammatory response as part of the immune response to stress, we decided to quantify the salivary levels of pro-inflammatory cytokines both before and after exposure to stress. We selected those cytokines that had previously shown changes in relation to stress in prior studies, such as IL-6 (1, 11, 18, 21, 27), whose variations showed controversy in different studies, such as IL-1β (18, 21, 33–36) and other inflammatory mediators, such as the acute-phase protein CRP, regarding which there is limited literature with controversy on the subject (18, 30, 36, 37).

Although there is considerable variability in the procedures of studies analyzing stress and inflammation through salivary biomarkers, there is consensus in accepting that circadian variations occur in the levels of both cortisol and certain inflammatory biomarkers. Therefore, we decided to avoid the first hour after awakening as the starting point of the study, thus eliminating the possibility that the physiological increase in cortisol at this time of day (12, 38) could influence the cortisol quantification and, similarly, avoided the circadian influence on IL-6 levels (39) whose peak release is traditionally assumed to occur between 7:00 PM and 5:00 AM (40). Salivary collection was conducted between 9:00 AM and 10:30 AM to avoid the first hour after awakening, which could alter cortisol concentrations. Upon arrival at their workstations, the first saliva sample was collected using the passive technique of Navazesh (41) in 2 ml cryovials and devices in Saliva Collection Aid (Salimetrics LLC, bioNova Científica S.L., Madrid, Spain), just before the start of the oral surgery procedure (pre). After the surgery was completed, a second saliva sample was collected during the hour after oral surgery performance (post).

The stressor selected was the performance of a dental implant placement surgery (from 1 to 3 implants) without other surgical procedures that would imply greater complexity. All surgeries were performed with an open flap technique, using the same drilling protocol, implant placement, and later simple flap suturing. In order to homogenize the surgery characteristics, all surgery procedures lasted between 45 and 60 min. No intraoperative complications arose in any of the cases.

Prior to the collection of the salivary samples on the study day, the students completed the perceived stress scale (PSS) described by Cohen et al. (42). The PSS is a 14-item instrument designed to measure the degree to which life situations are considered stressful, designed to explore the extent to which respondents found their lives unpredictable, uncontrollable, and overwhelming. The scale also includes a series of direct questions about the current levels of stress experienced. The perceived stress quantitative variable was recoded into a categorical variable as never or almost never stressed, occasionally stressed or often stressed, according to the cut-off points used by Torres-Lagunas et al. (43).

### 2.3 Biomarkers assessment

The saliva samples were centrifuged for 15 min at 1500 rpm immediately after collection in the Choukroun Duo Quattro (Nice, France) PRF centrifuge, and then frozen at −20 degrees Celsius within 4 h of collection, until storage at −80 degrees Celsius in the Thermo Scientific™ Revco Elite Plus ultra-low freezer (model ULT2586-6-D48, Thermo Fisher Scientific Inc., Massachusetts, USA), at the laboratories of the Complutense University of Madrid. The saliva samples were used for enzyme-linked immunosorbent assay (ELISA) techniques to quantify the concentration of several molecules using commercial kits for CRP, IL-1β, IL-6, and cortisol (Salimetrics Inc., State College, PA, USA), following the manufacturer’s instructions.

Briefly, to perform ELISA’s assay, the samples were completely thawed, vortexed, and centrifuged at 1500 *g* for 15 min in a 64R high-speed refrigerated centrifuge (Allegra^®^, Beckman Coulter Inc., Crea, California, USA). The supernatant was used for quantification, and except for cortisol, all saliva samples needed to be diluted: 2 times for CRP, 5 times for IL-6, and 15 times for IL-1β. The absorbance of the plates was measured using an iMark™ microplate absorbance reader (Bio-Rad, Hercules, California, USA) with a 450 nm filter and another correction filter at 620 nm. The concentrations for each molecule studied were calculated using the standard curves built from each assay and were expressed in pg/ml. The intra-assay coefficient of variation ranged from 2 to 4.8%, while the inter-assay coefficient of variation ranged from 2.6 to 6.6% (Table 1). A variable was created to assess the pre-post difference between for the four measured biomarkers.

**TABLE 1:** Coefficient of variation of biomarkers assessed in the research.

	Coefficient of variation	
	Intra-assay	Inter-assay
PCR	3.20%	2.60%
IL-1β	2%	4.50%
IL-6	4.80%	6.60%
Cortisol	4.60%	6%

### 2.4 Data analysis

A descriptive analysis was performed reporting the mean, standard deviation (SD), maximum, and minimum values for quantitative variables. For qualitative variables, frequency and percentage values were calculated. Normality analyses were conducted using the Kolmogorov-Smirnov test, although due to the small sample size non-parametric tests were performed. The Wilcoxon signed-rank test was applied to related samples (pre-post) to assess whether significant differences existed between measurements. To study potential significant differences between categorical variables, the Mann-Whitney U test was performed. The Spearman rank correlation coefficient was used to examine the association between quantitative variables. All tests were performed bilaterally with a significance level set at 95% (*p* < 0.05). The statistical software used for the analysis was SPSS (version 29.0.1.0, IBM, Armonk, NY, USA).

## 3 Results

### 3.1 Sample description

The total analyzed sample included 21 subjects, of whom 52.4% were female and 47.6% were male, with a mean age of 25.5 years (Table 2). About academic year distribution, 57.1% were first-year students, and 42.9% were in their second year. Smoking habits were also recorded, with 71.4% of participants identified as non-smokers and 28.6% as smokers. Additionally, perceived stress was evaluated, finding that 61.9% of respondents reported being occasionally stressed, 28.6% rarely or never perceived stress, and only 9.5% often experienced stress.

**TABLE 2:** Descriptive analysis of qualitative variables.

Variable	Categories	*n*	%
Gender	Male	10	47.6
	Female	11	52.4
Academic course	First-year	12	57.1
	Second-year	9	42.9
Smoker status	No	15	71.4
	Yes	6	28.6
Perceived stress	Never or almost never stressed	6	28.6
	Occasionally stressed	13	61.9
	Frequently stressed	2	9.5

The descriptive statistics of the dependent variables measured in the study were calculated (Table 3). Additionally, normality tests were performed, revealing that variables such as Cortisol pre, Cortisol difference, CRP-Post, IL-1β pre, IL-1β post, IL-1β difference, and IL-6 difference followed a normal distribution (Kolmogorov-Smirnov *p* > 0.05). However, based on the results, the hypothesis of normality can be rejected for the rest of the variables.

**TABLE 3:** Descriptive analysis of dependent variables and normality testing.

Variable	Mean	SD	Minimum	Maximum	Kolmogorov-Smirnov test *P*-value
Cortisol pre	398.5	246.1	63.6	999.1	0.109
Cortisol post	290.5	250.9	51.7	882.3	<0.05*
Cortisol difference	−108.2	166.7	−501.6	168.5	0.200
PCR pre	81.9	78.9	13.7	366.4	<0.001**
PCR post	81.9	52.7	17.2	221.3	0.093
PCR difference	−0.04	61.6	−173.2	106.3	<0.001**
IL1 β pre	30.2	25.1	1.3	77.4	0.061
IL 1 β post	28.4	20.2	1.8	69.8	0.200
IL 1 β difference	−1.7	16.4	−45.5	25.8	0.184
IL 6 pre	2.4	1.6	1.3	7.9	<0.001**
IL6 post	3.1	2.5	0.7	11.9	<0.05*
IL6 difference	0.7	1.4	−1.8	4.1	0.200

SD. Standard deviation.

*Statistically significant at *p* < 0.05.

**Statistically significant at *p* < 0.001.

### 3.2 Analysis of pre-post differences in dependent variables

The results indicate a significant decrease in cortisol levels between the pre- and post-measurements (mean difference = −108.2 ± 166.7) (Table 4). Conversely, IL-6 levels showed a significant increase in the post-measurement period (mean difference = 0.7 ± 1.4).

**TABLE 4:** Pre-post comparison values in analyzed biomarkers with Wilcoxon test.

Variables	*Z*	*p*
Cortisol – Pre; Cortisol – Post	−2.624	<0.05*
PCR – Pre; PCR – Post	−1.199	0.230
IL 1 β – Pre; IL 1 β – Post	−0.156	0.876
IL 6 – Pre; IL 6 – Post	−2.068	<0.05*

*Statistically significant at *p* < 0.05.

The influence of academic year, gender, or smoking status on the dependent variables was analyzed, and none were found to have a significant impact (Table 5).

**TABLE 5:** Signification for U Mann-Whitney test in the analyzed biomarkers according to qualitative variables.

	Cortisol difference		PCR difference		IL-1β difference		IL-6 difference	
	U Mann-Whitney	*P*-value	U Mann-Whitney	*P*-value	U Mann-Whitney	Sig	U Mann-Whitney	*P*-value
Academic course	47	0.619	44	0.477	47	0.619	41	0.356
Gender	44	0.439	53	0.888	45	0.481	47	0.573
Smoking status	39	0.640	33	0.350	36	0.484	37	0.533

### 3.3 Analysis of relationship between dependent variables

**FIGURE 1:**
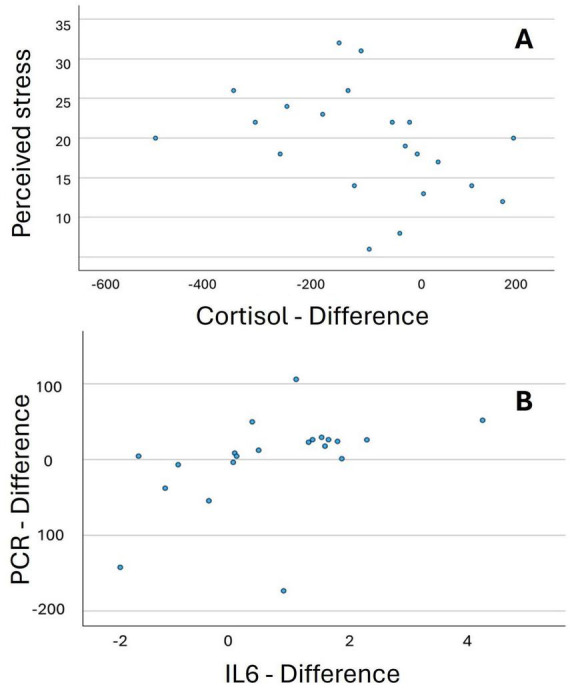
Scatter plot: relationship between perceived stress and cortisol difference **(A)**, and between IL6 difference and PCR difference **(B)**.

According to Spearman correlation results (Table 6), a strong positive monotonic relationship exists, where low cortisol levels are associated with high perceived stress levels (Figure 1A). Similarly, a moderate positive monotonic relationship was seen, where high CRP levels tend to correspond to high IL-6 values (Figure 1B).

**TABLE 6:** Spearman correlation results.

Spearman correlation	Cortisol difference	PCR difference	IL-1β difference	IL-6 difference	Perceived stress
Cortisol difference	1	0.326	−0.026	−0.040	−0.521*
PCR difference	–	1	0.222	0.631**	−0.196
IL-1β difference	–	–	1	−0.077	−0.050
IL-6 difference	–	–	–	1	0.174
Perceived stress	–	–	–	–	1

*Statistically significant at *p* < 0.05.

**Statistically significant at *p* < 0.001.

## 4 Discussion

A total of 21 subjects were studied, similar to Thompson et al. (36), with a sample size of 24 participants evaluating the same salivary biomarkers (cortisol, IL-1β, IL-6, and CRP) in a young population (aged 23 to 35 years) in their early professional practice. The aim of the study was to assess whether exposure to surgical procedures, which will be routine in their daily work as oral surgeons, generates measurable stress using biomarkers and whether this stress could have implications for their immune system, particularly through the activation of inflammation. The remaining studies compared in this work present highly variable sample sizes, with an average of 64.14 ± 44.35. The studies by Campisi et al. (37) and Dias et al. (33) had smallest number of participants (15 and 16 participants, respectively) and the highest, the studies by Goetz and Lucas (30), Hackett et al. (27) and Chiappelli et al. (11) (118, 140 y 150 participants, respectively). We conducted our study in Spain, with the entire sample consisting of European subjects. Most of the studies analyzed in this work were conducted in the United States (1, 11, 30, 35–37, 44), although another research has been conducted in Europe (18, 32), Brazil (33), or Asia (45). Some research does not specify subjects precedence (21, 27, 34, 46).

Regarding the methodology, salivary samples in our study were collected while avoiding the first hour after waking to prevent the circadian cortisol peak that occurs during that time of day, following the approach used by Goetz and Lucas (30). Although some authors collect samples after midday or in the afternoon (1, 21, 46), in our case, this was also excluded to avoid variations in IL-6 levels due to circadian rhythms (39). The collection was performed using Navazesh’s passive technique with unstimulated saliva, following the same method as described by previous authors (1, 11, 30, 34–36, 44) since other methods, such as collection using cotton swabs (18, 21, 33, 45, 46) may affect the determination of the salivary cortisol concentration (47).

In relation to the alteration of salivary biomarkers in response to stress and their consequent influence on the immune system, several factors must be considered. First, the nature of the stressor, we considered cognitive-type stressors, excluding physical stressors (1, 11, 18, 21, 27, 30, 33–37, 44, 46). Additionally, the circadian rhythms of cortisol must be considered (16). The timing of exposure to the laboratory stressor, therefore, influences the cortisol response, with higher levels in the morning than in the afternoon (48, 49). It is also important to highlight the recovery time to baseline levels of biomarkers, with cortisol recovery, after reaching its peak following the onset of an acute stressor, taking 60 to 70 min (3).

There are multiple studies showing that there is an alteration in the salivary cortisol concentration after exposing an individual to an acute stressor. According to our results, a decrease in cortisol levels occurs once the stressor has finished, in agreement with the study conducted by Hackett et al. (27) a decrease in cortisol levels following exposure to an acute stressor, although marked interindividual differences have been proved. It was noted that the rate of cortisol decline in saliva was more rapid 20 min after the stressor, compared to at once post-stressor. Similarly, Thompson et al. (36), after subjecting the study participants to sleep deprivation for one night, they also found a decrease in salivary cortisol concentrations compared to the baseline concentrations without sleep deprivation.

On the other hand, some authors suggest that there is an increase in salivary cortisol concentrations after the stressor has been overcome (11, 32, 33, 35, 37). It is worth noting that in the studies conducted by Kennedy et al. (32) and Chiappelli et al. (11) in part of the sample, there were concomitant health conditions (irritable bowel syndrome and schizophrenia, respectively), as opposed to our sample, which consisted of healthy individuals without systemic conditions that could affect cortisol release. Días and Scalabrini-Neto (33) used devices known as cotton swabs for salivary sample collection, while in our case, we opted for the passive collection technique, because authors have stated that cotton swabs may alter the collection of salivary cortisol (47).

One of the goals of our study was to assess whether the stress system undergoes habituation after exposing different subjects to the same stressor. To this end, we compared first-year students with second-year students, the latter having been exposed to the stressor more often. No statistically significant differences were found between the two groups. However, authors such as Lester et al. (1) and such habituation when assessing the cortisol response during a university examination period. In our study, the habitual cortisol release of the subjects without exposure to external stressors was not previously assessed, and therefore, we do not know the baseline concentrations typically released by them. Studies show that subjects who habitually show a lower cortisol response tend to have higher cytokine responses (27).

Regarding IL-6, in our study as in earlier studies (1, 11, 18, 21, 27, 36). There is an increase in IL-6 following exposure to the stressor. It is worth noting that, unlike our study, in which no statistically significant differences were found between male and female, Maydych et al. (21) find higher salivary IL-6 levels in men compared to women after exposure to the PASAT stressor. On the other hand, Chiappelli et al. (11) indicates that more intense cortisol responses inhibit the later pro-inflammatory responses. Therefore, it is expected that higher cortisol levels would result in a smaller increase in IL-6. In the study by La Fratta et al. (18) obtain results like ours about the inflammatory response, as both plasma and salivary IL-6 levels show an opposite trend compared to other cytokines analyzed, such as IL-1β.

In relation to IL-1β no statistically significant differences were found in IL-1β concentrations pre- and post-stress, similar to the results obtained by Thompson et al. (36). There is discrepancy regarding the behavior of this cytokine in saliva following acute stress, as some studies (21, 36, 45, 46) stated that the levels of this cytokine significantly increase after stress, while others found lower concentrations of this cytokine after the stressor compared to baseline (18). Shields et al. (35) relates these findings to those of other studies that support the idea that salivary inflammatory mediator levels decrease following exposure to pain-based stressors.

There are a limited number of studies that evaluate the behavior of CRP after activation of the stress system, particularly when assessed as a salivary biomarker. Our study shows no changes in salivary CRP levels after comparing pre- and post-stressor concentrations, similar to previous studies (18, 32, 37). On the contrary, both the study by Goetz and Lucas (30) and Thompson et al. (36), found a statistically significant increase when comparing salivary levels of this protein at baseline and after exposing the study subjects to a stressor. It is worth noting the predictable association observed in our study between the biomarkers CRP and IL-6, such that an increase in CRP levels tends to be accompanied by an increase in IL-6 levels, which is explained by the fact that CRP release depends on the action of IL-6 (28, 50).

The neuroendocrine system’s response to stressors will, in part, depend on the intensity with which the stressor is perceived by the subject. In our study, we postulated that higher perceived stress would be associated with higher cortisol concentrations, yielding the result that there is a correlation between both values, such that high cortisol levels are accompanied by high perceived stress levels. In contrast, some studies (46) found an inverse relationship, where higher perceived stress was associated with lower cortisol concentrations. Our study has limitations that should be considered when interpreting the data. One key limitation is the small sample size, as well as the need for a more extended assessment of the study subjects in terms of biomarkers. This fact, and the absence of basal determination of salivary stress levels, could decrease the generalizability of the results and the interpretation of changes as purely stress-induced. Also, as the research has been conducted on a single session of surgery the variation in clinical stress over time could not be assessed. On the other hand, the research has the strength of evaluating the novel application of non-invasive salivary biomarkers, enabling the concurrent measurement of stress and inflammatory response in an actual clinical environment, thereby providing insight into the physiological effect of dental training on postgraduate students.

Future research would benefit from analyzing biomarker concentrations over a longer period, both before and after the study, to understand their behavior under different variables (sleep quality, circadian rhythms, daily life stressors, hormonal cycles, etc.), as well as the moments of variation in concentrations. However, the fact that we analyzed more than one inflammatory biomarker with different behaviors has allowed us to assess whether there is a relationship between them in terms of the stress response. Furthermore, by using a sample of healthy individuals, we ensured that no other health conditions influenced the release of biomarkers. In future research, we aim to increase the sample size, perform longitudinal biomarker analyses, incorporate various types of dental procedures, and include participants from additional postgraduate programs. This will allow to better assess real changes in salivary biomarkers associated with stress in students performing complex clinical procedures.

## 5 Conclusion

Considering the aforementioned limitations, the findings indicate a significant decrease in salivary cortisol (stress) levels and a significant increase in salivary IL-6 levels following a dental implantology procedure in postgraduate oral surgery students. Academic year, gender, and smoking status did not influence the salivary stress and inflammatory biomarkers. A negative correlation was seen between cortisol salivary levels and perceived stress, while a positive correlation was found between cortisol and IL-6 levels.

## Data Availability

The raw data supporting the conclusions of this article will be made available by the authors, without undue reservation.
